# Impact of Rapid Molecular Diagnostic Testing on Outcomes of Patients With Vancomycin-Resistant Enterococcal Bacteremia

**DOI:** 10.1093/ofid/ofaf757

**Published:** 2025-12-12

**Authors:** Michael R Hovan, Michael J Burkitt, Sierra A Derti, Judith U Hargrave, Angela S De Cordova, Matthew S Simon, Stephen G Jenkins, Lars F Westblade, Michael J Satlin

**Affiliations:** Department of Medicine, Division of Pulmonary & Critical Care Medicine, NewYork-Presbyterian/Weill Cornell Medicine, New York, New York, USA; Pittsburgh Infectious Diseases, LTD., Pittsburgh, Pennsylvania, USA; Department of Medicine, Division of Internal Medicine, Allegheny Health Network, Pittsburgh, Pennsylvania, USA; Clinical Microbiology Service, NewYork-Presbyterian/Weill Cornell Medical Center, New York, New York, USA; Department of Pharmacy, NewYork-Presbyterian/Weill Cornell Medical Center, New York, New York, USA; Department of Medicine, Division of Infectious Diseases, Weill Cornell Medicine, New York, New York, USA; Department of Medicine, Division of Infectious Diseases, Weill Cornell Medicine, New York, New York, USA; Department of Pathology and Laboratory Medicine, Weill Cornell Medicine, New York, New York, USA; Department of Medicine, Division of Infectious Diseases, Weill Cornell Medicine, New York, New York, USA; Department of Pathology and Laboratory Medicine, Weill Cornell Medicine, New York, New York, USA; Department of Pediatrics, Weill Cornell Medicine, New York, New York, USA; Department of Medicine, Division of Infectious Diseases, Weill Cornell Medicine, New York, New York, USA; Department of Pathology and Laboratory Medicine, Weill Cornell Medicine, New York, New York, USA

## Abstract

**Background:**

Vancomycin-resistant enterococci (VRE) bacteremia is associated with substantial mortality. Rapid molecular diagnostic testing (RMDT) that detects enterococci and vancomycin resistance genes from positive blood culture broths may lead to earlier appropriate antimicrobial therapy for VRE bacteremia, which could improve clinical outcomes.

**Methods:**

We conducted a retrospective cohort study of patients with VRE bacteremia from 2010 to 2019. RMDT that detects enterococci and vancomycin resistance genes from positive blood cultures was implemented in October 2014. We compared time to active antimicrobial administration, mortality, and other outcomes in patients whose positive blood cultures underwent RMDT (postintervention) and those whose did not (preintervention).

**Results:**

Of 577 eligible patients with VRE bacteremia, 237 (41.1%) had blood cultures that underwent RMDT. The RMDT cohort had shorter median time from culture collection until receipt of active antimicrobial therapy (21 vs 32 hours, *P* < .001) than the non-RMDT cohort. There was no difference in 30-day mortality (31.6% vs 36.5%, *P* = .230). A post hoc subgroup analysis excluding patients with leukemia (who received empiric VRE-active therapy based on Gram stain results) found that RMDT was associated with decreased 30-day mortality: 29.6% versus 40.8%, *P* = .037. On multivariate analysis, that improvement in 30-day mortality in patients without leukemia did not persist.

**Conclusions:**

RMDT was associated with decreased time to receipt of active antimicrobial therapy in VRE bacteremia. There was no improvement in mortality associated with the use of RMDT. RMDT for VRE bacteremia may improve time to identification and treatment of VRE bacteremia but does not clearly improve clinical outcomes.

## Teaser

Rapid molecular diagnostic testing was associated with decreased time to active antimicrobial therapy in vancomycin-resistant enterococcal bacteremia and improved 30-day mortality in patients without leukemia but not in the overall cohort.

Infections due to vancomycin-resistant enterococci (VRE) are associated with significant morbidity and mortality and pose a significant public health threat in the United States and globally [1]. The US Centers for Disease Control and Prevention (CDC) classifies VRE at a threat level of “serious,” the second highest antimicrobial resistance threat level that the CDC assigns [2, 3]. Vancomycin-resistant enterococci bacteremia has a 28-day mortality rate ranging from 33.7% to 50.5% [4–6], which is 1.8–2.4 times greater than in those with bacteremia due to vancomycin-susceptible *Enterococcus* spp [7]. VRE also frequently cause bacteremia in immunocompromised patients, such as those with hematologic malignancies and those undergoing hematopoietic stem cell transplantation [8–13].

Modeling studies have shown that a delay in appropriate antimicrobial therapy of more than 48 hours for VRE bacteremia is associated with a 3-fold increase in 30-day mortality [14]. Moreover, a multicenter study from Italy showed that the administration of effective antibiotic therapy within 48 hours from blood culture collection was associated with decreased 30-day mortality rates [15].

Multiple rapid molecular diagnostic tests (RMDT) have become available that can be applied to positive blood culture broths to detect the *vanA/B* gene that confers vancomycin resistance in enterococci. These assays can detect VRE within 2–3 hours after blood culture positivity, 24–72 hours before the availability of antimicrobial susceptibility testing results from isolated colonies [16, 17]. Despite the potential for these rapid molecular diagnostic tests to improve outcomes of patients with VRE bacteremia by leading to faster initiation of effective therapy [18, 19], few studies have reported the clinical impact of these tests for VRE bacteremia. These observational studies have shown that RMDT use in VRE bacteremia is associated with decreased time to identification of vancomycin resistance and initiation of effective antimicrobial therapy but no improvement in mortality, possibly due to small sample sizes and limited power to detect differences in clinical outcomes [18–21].

Given the limitations of current data regarding the impact of RMDT on clinical outcomes with VRE bacteremia, we reviewed our experience before and after the implementation of RMDT that detect *Enterococcus* species and *vanA/B* directly from positive blood culture broths. Our primary objective was to determine if the use of RMDT that detects VRE directly from positive blood culture broths was associated with improved clinical outcomes in patients with VRE bacteremia.

## METHODS

### Study Design and Microbiologic Testing

We performed a retrospective, preintervention/postintervention study of patients with VRE bacteremia between 20 September 2010 and 31 December 2019 at NewYork-Presbyterian/Weill Cornell Medical Center, an 862-bed academic medical center in New York, NY. The study was approved by the Weill Cornell Medicine Institutional Review Board (#20-02021550) with a waiver of informed consent. RMDT use at our institution began on 14 October 2014. Two different testing platforms were used during the study: the Verigene Gram-Positive Blood Culture Nucleic Acid Test (BC-GP; Luminex Corp., Austin, TX) and the FilmArray Blood Culture Identification Panel (BCID, BioFire Diagnostics, LLC., Salt Lake City, UT) [16, 17]. The Verigene platform and BCID platform were both used during the study period. The Verigene BC-GP Panel identified *Enterococcus faecalis* and *Enterococcus faecium*, as well as *vanA* and *vanB*. The BCID Panel identified *Enterococcus* species, as well as *vanA* and *vanB*, but did not differentiate between *E. faecalis* and *E. faecium*. Any positive blood cultures for VRE that did not undergo RMDT were included in the no RMDT group. Vancomycin resistance was defined as a minimum inhibitory concentration of ≥32 µg/mL, in accordance with breakpoints of the Clinical and Laboratory Standards Institute [22]. Organism identification and antimicrobial susceptibility testing were performed using conventional clinical microbiology methods.

Antimicrobial stewardship program (ASP) activities included prior authorization for agents with activity against VRE (unchanged during the entire study period) and postprescription review with feedback, initiated in 2016. Beginning in 2018, hospital-wide postprescription review activities included daily (Monday to Friday) infectious diseases (ID) pharmacist review of positive blood cultures via surveillance reports, but not immediate notification of positive blood cultures to the ID pharmacist. Additionally, as part of RMDT implementation, institutional guidelines for result interpretation and antimicrobial therapy were developed and published on the hospital intranet in December 2014, and updated annually thereafter.

### Patient Cohort and Study Outcomes

All adult patients with their first episode of VRE bacteremia during the study period were included for analysis unless they met an exclusion criterion. Additional episodes of VRE bacteremia in the same patient within the study period were not included. Exclusion criteria included blood cultures positive for VRE that were obtained during outpatient care or at a different hospital, patients who left against medical advice prior to initiation of antimicrobial therapy, patients who died or who pursued only comfort measures prior to blood culture positivity, and patients whose VRE positive blood cultures were deemed to be contaminants by the patient care team.

Clinical data were collected from the electronic medical record by an ID physician (M. J. B.), including demographics, history of infection or colonization with VRE, Charlson Comorbidity Index, and Pitt Bacteremia Score [22, 23]. We also recorded time from blood culture collection to detection of vancomycin resistance (detection of *vanA* or *vanB* and *Enterococcus* species or detection of VRE by conventional culture and antimicrobial susceptibility testing), antimicrobial therapies, and time until receipt of active therapy (defined as an agent to which the VRE isolated in blood culture tested susceptible). Bacteremia onset was defined as the time of collection of the index blood culture positive for VRE. The primary outcome was 30-day in-hospital all-cause mortality from bacteremia onset, based on data available in our hospital system's electronic medical records. Secondary outcomes included microbiologic failure (composite outcome of death within 96 hours or persistently positive blood cultures drawn greater than 96 hours after the index culture), time to blood culture clearance (defined as the time to collection of the first of two consecutive negative blood cultures), in-hospital mortality, hospital length of stay, hospital readmission within 30 days, and recurrence of VRE bacteremia within 90 days.

### Post hoc Analysis

As we reviewed the electronic medical records, we noted that patients with leukemia were initiated on VRE-active therapy if Gram-positive cocci in pairs and/or short chains were identified on Gram stain of the positive blood culture broths throughout the study period. We hypothesized that this empiric coverage of VRE may have decreased any potential clinical benefit of the RMDT test. We therefore performed a post hoc subgroup analysis for 30-day mortality in patients with and without leukemia and other related subgroups, such as patients with neutropenia.

### Statistical Analyses

Data were analyzed using Stata/MP Version 17.0 (StataCorp LLC, College Station, TX). Continuous data were reported as medians with interquartile ranges (IQR). Categorical data were reported as percentages. The Wilcoxon rank-sum test was used for the comparison of continuous variables. The χ^2^ test was used for the comparison of categorical variables. The significance level was set at a two-sided *P* value of <.05. We performed a time-to-event analysis using a Cox proportional hazard model to estimate the effect of RMDT status on the hazard ratio of in-hospital mortality at 30 days after bacteremia onset. For our multivariate analyses, we included the following baseline variables that we determined were important to adjust for in determining the relationship between RMDT and our primary outcome of 30-day mortality: age, race, Charlson Comorbidity Index and Pitt Bacteremia Scores, and severe neutropenia.

## RESULTS

### Study Cohort and Baseline Characteristics

**Figure 1.**
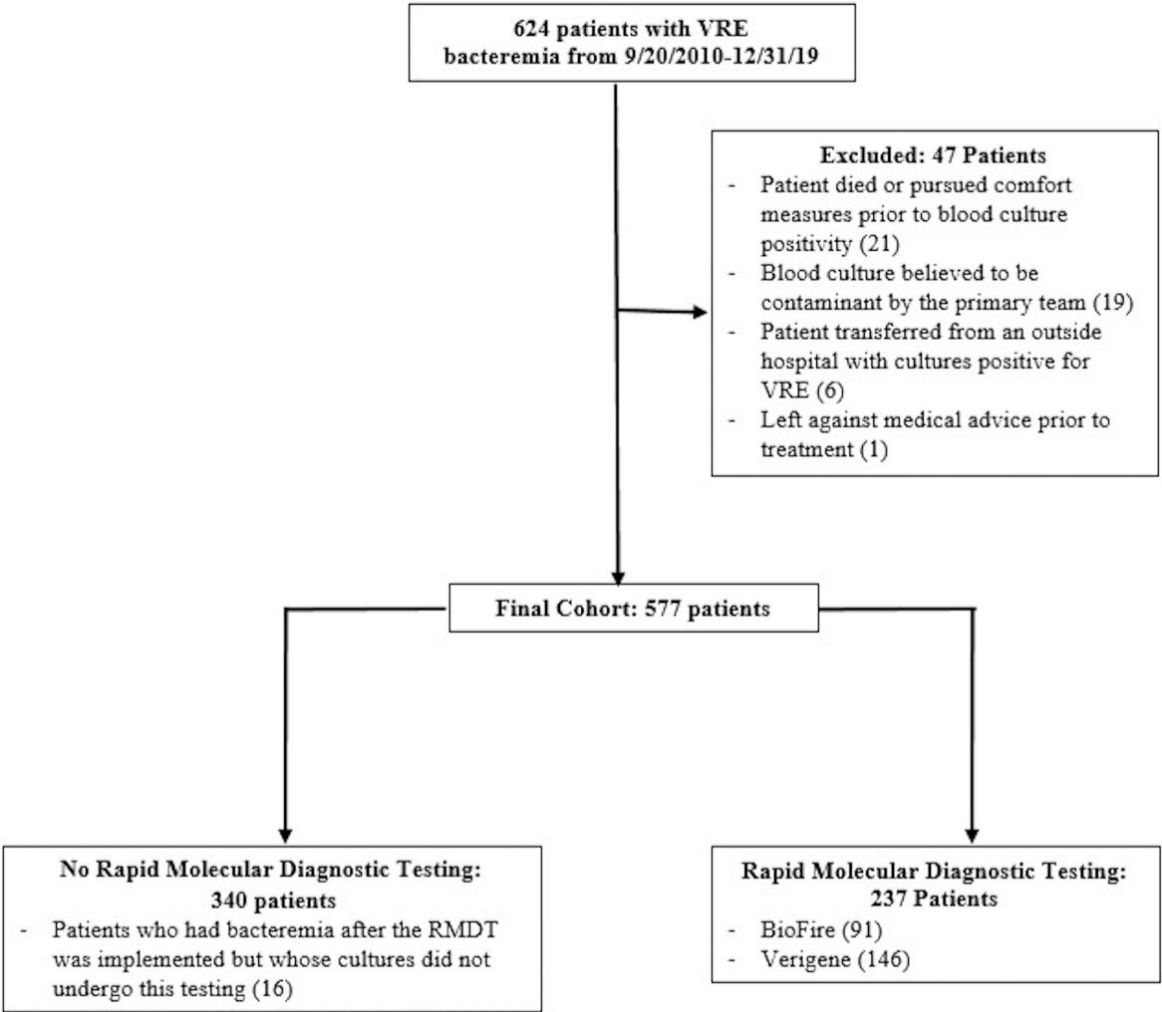
Flowchart of study population identification, inclusion, and exclusion criteria.

There were 624 patients with blood cultures positive for VRE during the study period. Of these, 47 were excluded, most commonly because the patient died or pursued comfort measures prior to blood culture positivity or because the blood cultures were considered contaminants by the primary medical team (Figure 1). Five hundred seventy-seven patients were included in our final cohort, of which 237 (41.1%) had blood cultures that underwent RMDT and 340 (58.9%) did not.

The median age of the cohort was 65 years, 43.4% were female, and the most common comorbidity was leukemia (43.5%). One hundred fifty-one (26.2%) patients had a history of VRE infection or colonization and the most common source of bacteremia was gut translocation in the setting of chemotherapy-induced neutropenia (46.4%). Patients in the RMDT group were more likely to have chronic obstructive pulmonary disease compared to those in the no RMDT group (11.4% vs 5.2%, *P* = .007; Table 1) as well as liver disease (5.9% vs 2.1%, *P* = .015; Table 1). There were no significant differences between the 2 groups in terms of age, comorbidities, Charlson Comorbidity Index, or rates of severe neutropenia (Table 1). There were also no significant differences in the baseline clinical characteristics of both groups during the initial 48 hours following collection of the index blood culture including Pitt Bacteremia Score or admission to an ICU. One patient had an allergy to daptomycin, and none had an allergy to linezolid.

**Table 1.** Comparison of Baseline Demographic and Clinical and Microbiologic Characteristics Between Study Groups

Variable	RMDT (n = 237)	No RMDT (n = 340)	*P* value
Demographics			
Age (median, IQR)	66 (56–74)	65 (54–74)	.678
Female	103 (43.4)	147 (43.2)	.957
Race			<.001
White	111 (46.8)	139 (40.9)	
Black or African American	39 (16.5)	22 (6.5)	
Asian	18 (7.6)	20 (5.9)	
Native Hawaiian or other Pacific islander	1 (0.4)	2 (0.6)	
Other or unknown	68 (28.7)	157 (46.1)	
Comorbidities			
Leukemia	95 (40.1)	156 (45.9)	.167
Solid organ malignancy	50 (21.1)	75 (22.1)	.783
Diabetes mellitus	52 (21.9)	67 (19.7)	.514
Lymphoma	33 (13.9)	51 (15.0)	.718
Chronic kidney disease	31 (13.1)	31 (9.1)	.131
Congestive heart failure	27 (11.4)	31 (9.1)	.371
Chronic obstructive pulmonary disease	27 (11.4)	18 (5.2)	.007
Cerebrovascular accident	15 (6.3)	24 (7.1)	.731
Myocardial infarction	14 (5.9)	14 (4.1)	.325
Peptic ulcer disease	13 (5.5)	14 (4.1)	.444
Peripheral vascular disease	11 (4.6)	13 (3.8)	.628
Dementia	8 (3.4)	14 (4.1)	.647
Connective tissue disease	8 (3.4)	11 (3.2)	1.000
Liver disease	14 (5.9)	7 (2.1)	.015
Hemiplegia	8 (3.4)	5 (1.5)	.158
Human immunodeficiency virus infection	5 (2.1)	17 (5.0)	.081
Charlson Comorbidity Index (median, IQR)	2 (2–4)	2 (2–4)	.823
Severe Neutropenia (ANC <500 cells/µL)	115 (41.5)	170 (46.0)	.460
Known prior history of VRE (infection or colonization)	68 (28.7)	83 (24.4)	.250
Baseline Clinical Characteristics			
Pitt Bacteremia Score (median, IQR)	1 (0–3)	1 (0–3)	.533
In intensive care unit at time of bacteremia onset	55 (23.2)	101 (29.7)	.084
Receiving vancomycin at time of bacteremia onset	50 (21.1)	91 (26.7)	.119
Source^a^			
Central venous catheter	13 (5.5)	14 (4.1)	.399
Gastrointestinal translocation	110 (46.4)	158 (46.4)	.989
Intra-abdominal	28 (11.8)	36 (10.6)	.644
Skin	4 (1.7)	4 (1.2)	.605
Urine	12 (5.1)	11 (3.2)	.283
Other/unidentified	70 (29.5)	117 (34.4)	.218
Baseline microbiologic characteristics			
*Enterococcus faecium*	221 (93.2)	320 (94.1)	.841
*Enterococcus faecalis*	15 (6.3)	18 (5.3)	
Polymicrobial	54 (22.8)	82 (24.1)	.711
Susceptibilities			
Ampicillin susceptible	13 (5.5)	18 (5.3)	.920
Daptomycin susceptible	212 (89.5)	315 (92.6)	.179
Linezolid susceptible	223 (94.1)	223 (93.2)	.679

Results listed as n (% of total) unless otherwise noted.

^a^Source determined by review of the electronic medical record by an infectious disease physician.

### Microbiologic and Treatment Data

*Enterococcus faecium* was the most common bloodstream pathogen in both groups (RMDT group: 93.2%; no RMDT group: 94.1%; Table 1). Similar proportions of blood cultures were polymicrobial (22.8% in the RMDT group vs 24.1% in the no RMDT group). The percentages of bloodstream isolates that were susceptible to daptomycin (89.5% in RMDT group, 92.6% in the no RMDT group) and linezolid (94.1% in RMDT group, 93.2% in no RMDT group) were also similar.

Use of RMDT was associated with decreased time to detection of VRE (20 hours [IQR 17–22] vs 62 hours [IQR 54–69], *P* < .001) and decreased time to administration of active antibiotic therapy from bacteremia onset (21 hours [IQR 17–25] vs 32 hours [IQR 21–59], *P* < .001, Table 2). Linezolid was the first active antimicrobial administered in 68.4% of patients in the RMDT group and 71.2% of patients in the no RMDT group (Supplementary Table 1). There were no significant differences in the proportions of patients who received daptomycin as an initial antimicrobial (RMDT: 27.0% vs no RMDT: 21.8%) or the dosage of daptomycin (RMDT: 6.54 mg/kg vs no RMDT: 6.83 mg/kg) between groups.

**Table 2.** Comparison of Clinical and Microbiological Outcomes Between Study Groups

Outcome	RMDT (n = 237)	No RMDT (n = 340)	*P* value
In-hospital mortality within 30 d of bacteremia onset (primary outcome)	75 (31.6)	124 (36.5)	.230
Time from blood culture collection to susceptibilities/detection of vancomycin-resistant enterococci (median hours, IQR)	20 (17–22)	62 (54–69)	<.001
Time from blood culture collection to administration of active antimicrobial therapy^a^ (median hours, IQR)	21 (17–25)	32 (21–59)	<.001
Microbiologic outcomes			
Microbiologic failure^b^	15 (6.3)	16 (4.7)	.395
Time to first negative blood culture (median hours, IQR)	43 (26–79)	53 (30–97)	.032
Follow-up blood cultures positive for VRE	60 (25.3)	96 (28.2)	.437
Other clinical outcomes			
Transfer to ICU within 7 d of positive culture^c^	25/182 (13.7)	34/239 (14.2)	.886
ID consult within 7 d of positive culture	188 (79.3)	250 (73.5)	.109
In-hospital mortality at any time	92 (38.8)	142 (41.8)	.478
Hospital length of stay postpositive culture (median days, IQR)	15.5 (1.6–86.4)	14.0 (1.5–71.1)	.165
Hospital readmission within 30 d of discharge	52 (21.9)	76 (22.4)	.907
Relapse of VRE bacteremia within 90 d	24 (10.1)	34 (10.0)	.960

All results listed as n (%) unless otherwise noted.

^a^Active antimicrobial therapy is defined as antimicrobials which the isolate was shown to be susceptible to on *in vitro* susceptibility testing.

^b^Death or persistently positive blood cultures at 96 h following collection of index blood culture.

^c^Denominator different as variable excludes patients already in ICU at time of bacteremia onset.

### Outcomes

**Figure 2.**
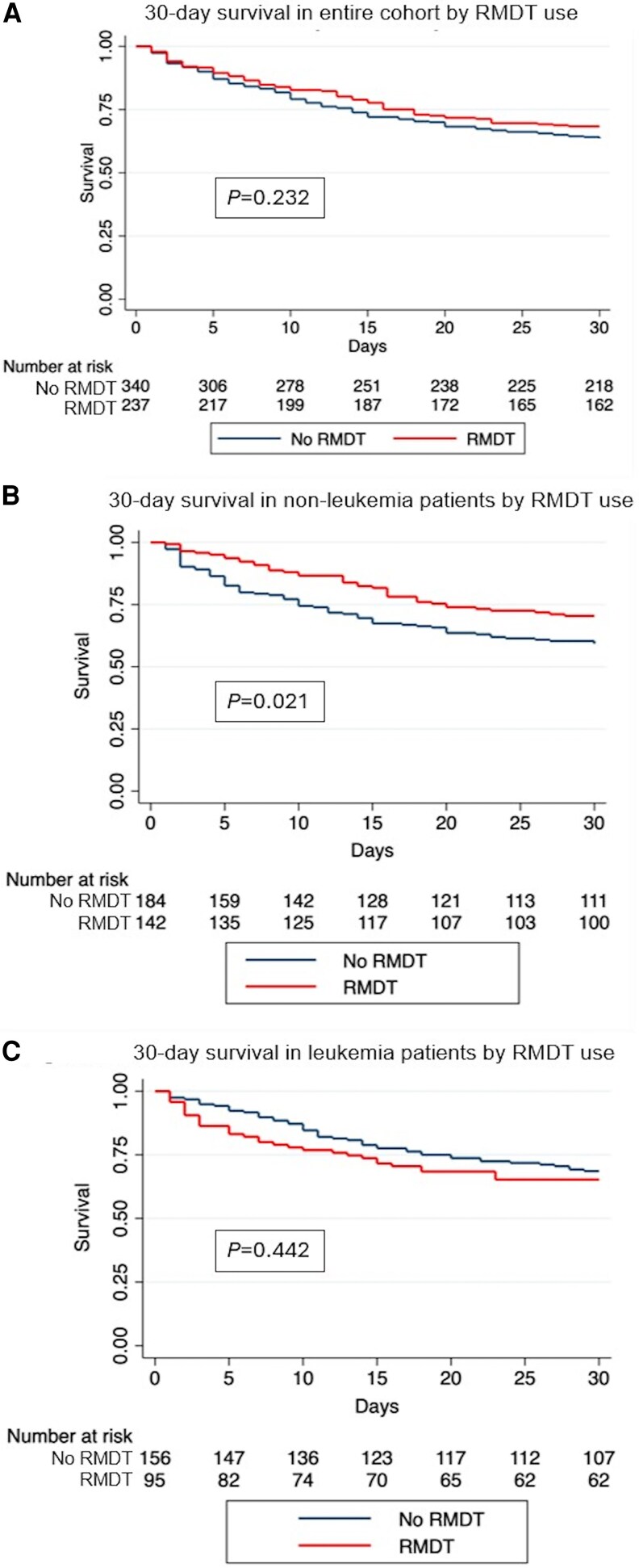
Kaplan–Meier curves, overall and by subgroup.

Seventy-five (31.6%) of 237 patients in the RMDT group and 124 (36.5%) of 340 patients in the no RMDT group died within 30 days of bacteremia onset (*P* = .230, Table 2). There was also no significant difference in in-hospital mortality between the 2 groups (RMDT: 38.8% vs no RMDT: 41.8%, *P* = .478). Kaplan–Meier analysis for 30-day mortality and a Cox proportional hazards model (hazard ratio 0.84, 95% confidence interval: 0.63, 1.12; *P* = .232) also did not show statistically significant differences between the RMDT and no RMDT group (Figure 2*A*).

RMDT was associated with decreased time to clearance of blood cultures (43 hours [IQR 26–79] vs 53 hours [IQR 30–97], *P* = .032, Table 2). However, there was no statistically significant difference in the composite outcome of death or microbiologic failure at 96 hours between the 2 groups (RMDT: 6.3% vs no RMDT: 4.7%, *P* = .395). There were also no significant differences in hospital readmission within 30 days of discharge, relapse of VRE bacteremia in 90 days following clearance of blood cultures, or transfer to ICU following development of bacteremia. On multivariate analysis, only increased Pitt Bacteremia score was independently associated with increased 30-day mortality among the entire cohort (Supplementary Table 3).

### Post hoc Subgroup Analysis Excluding Patients With Leukemia

Patients without leukemia had a greater decrease in the median time to effective antimicrobial administration between RMDT and no RDMT groups compared to patients with leukemia; without leukemia: RMDT: 22 hours versus no RMDT: 45 hours, *P* = <.001; with leukemia: RMDT: 21 hours versus no RMDT: 25 hours, *P* < .001. Other secondary outcomes stratified by leukemia status are presented in Supplementary Table 2.

**Figure 3.**
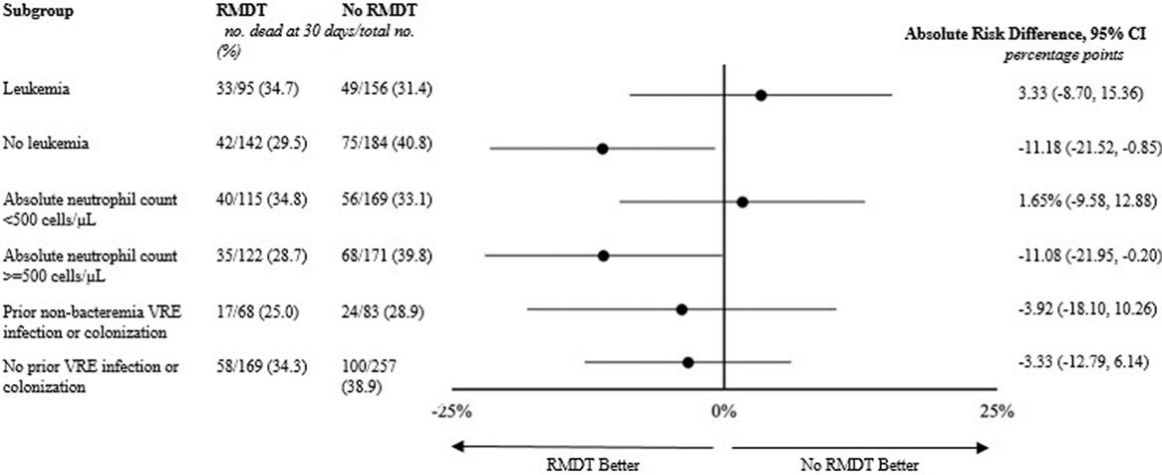
Subgroup analysis of absolute risk difference of 30-day mortality by RMDT use.

In patients without leukemia, those whose blood cultures underwent RMDT had lower 30-day mortality than those whose blood cultures did not undergo RMDT (No RMDT: 75/184, 40.8% vs RMDT: 42/142; 29.6, absolute risk difference: −11.2%, 95% CI −21.52, −0.85). In contrast, there was no significant difference in 30-day mortality in patients with leukemia (Figure 3). Kaplan–Meier curves comparing the use of RMDT in leukemia and nonleukemia patients are displayed in Figure 2*B* and 2*C*. In patients with leukemia, there was no significant difference between the 2 curves by Cox-regression (hazard ratio [HR] 1.19, 95% CI 0.77, 1.85, *P* = .442) while in patients without leukemia there was a decreased hazard of death at 30 days with the use of RMDT (HR 0.647, 95% CI 0.443, 0.944, *P* = .021). Similar differences were noted between patients without neutropenia, who had lower mortality with use of RMDT, and patients with neutropenia, who did not have lower mortality with use of RMDT.

A multivariate analysis was performed among patients without leukemia controlling for a number of baseline patient characteristics including age, race, Charlson Comorbidity Index, Pitt Bacteremia Score, and severe neutropenia (absolute neutrophil count [ANC] <500 cells/µL). When controlling for these characteristics, RMDT use was not associated with a statistically significant decreased odds of death at 30 days (adjusted odds ratio [aOR] = 0.71, 95% CI 0.42–1.18, Supplementary Table 4).

### Discussion

Our study examining the use of RMDT in VRE bacteremia showed that there was no significant difference in 30-day mortality with the use of RMDT compared to no RMDT in the overall cohort despite a shorter time to identification of VRE and shorter delays in administration of effective antimicrobial therapy. Moreover, there were no significant improvements in outcomes of clinical importance with the use of RMDT, including in-hospital mortality, hospital length of stay, or microbiologic failure. However, a post hoc subgroup analysis that excluded patients with leukemia, who were typically treated empirically for VRE based on Gram stain results, showed that RMDT use was associated with decreased 30-day mortality rates. However, RMDT was not associated with a statistically significant decrease in 30-day mortality in this subgroup when controlling for baseline characteristics.

This study supports prior reports that have shown that RMDT use decreases the time to identification of VRE in blood cultures and administration of effective antimicrobials [18–21]. This is unsurprising as conventional methods for microbial identification and susceptibility testing take several days to yield results, which can lead to significant delays in the administration of effective antibiotics. The earlier administration of effective targeted antimicrobial therapy would be expected to improve outcomes, as prior studies have shown the earlier administration of antimicrobials to be associated with decreased mortality in patients with sepsis and specifically in patients with VRE bacteremia [6, 12–20, 23–26]. However, our study did not demonstrate an improvement in 30-day mortality in our overall population.

We believe this lack of impact on mortality may partially be explained by the low threshold to initiate therapy active against VRE in the leukemia population at our institution, where the ID service recommended antibiotics with activity against VRE when blood cultures were positive with Gram stain revealing gram-positive cocci in pairs and/or short chains. This was reflected in only a 4-hour median difference in time to active therapy in patients with leukemia, compared to a 23-hour median difference in other patients. Moreover, we found that in a sensitivity analysis where we excluded patients with leukemia there was an 11% decrease in the absolute 30-day mortality risk with the use of RMDT. Although caution is warranted in interpreting results from a subgroup analysis that was not prespecified, we believe it is reasonable to hypothesize that the reduction in time to administration of effective antibiotics through use of the RMDT may have contributed to the mortality benefit observed in patients without leukemia. Alternatively, given the lack of statistical significance of the adjusted odds of mortality when controlling for baseline characteristics, it is also possible that other factors contributed to the decreased mortality observed with RMDT in patients without leukemia.

Our study has several limitations. First, it is a retrospective, observational, pre–post intervention study and is inherently at risk of bias. One bias could be changes in the management of VRE bacteremia other than the initiation of RMDT that occurred after the intervention over the study period of 10 years. For instance, the hospital ASP implemented postprescription review of positive blood culture treatment during the study period. However, these reviews initially focused on opportunities for de-escalation and occurred periodically (generally once daily on weekdays only and not immediately following positive RMDT results), and thus were unlikely to significantly modify treatment for VRE bacteremia. It is possible that with real-time alerts to hospital ASP with RMDT results, we could expect an even larger difference in outcomes than what was found as the combination of RMDT with ASP had led to greater benefit than the use of RMDT alone [27]. Moreover, we did not find differences in the choice of anti-VRE therapy or the dosage of daptomycin between study groups to suggest a change in the treatment of VRE bacteremia (Supplementary Table 1). There were no other identified treatment or infection control practices that changed over the course of the study period. Additionally, a large percentage of our study population had hematologic malignancies, which may limit generalizability to other populations. However, patients with hematologic malignancies and those undergoing stem cell transplantation are at high risk of VRE colonization and infection, so this finding is expected [8].

Despite these limitations, our study also has several strengths. First, this is the largest sample size published to date that addresses the use of RMDT in VRE bacteremia. Our study population is also well-balanced based on comorbidities and bacteremia severity. The overall mortality associated with VRE bacteremia in our cohort is also similar to that seen in prior studies, which suggests generalizability.

In conclusion, we found that RMDT for the identification of VRE was associated with decreased time to detection of vancomycin resistance and time to administration of effective antimicrobials in our overall population. While an improvement in 30-day mortality was seen in patients without leukemia or neutropenia, this improvement did not remain statistically significant when controlling for other baseline patient characteristics. This suggests that RMDT use may be associated with decreased mortality in patient populations where empiric anti-VRE therapy is often not considered.

## Supplementary Material

ofaf757_Supplementary_Data

## Supplementary Data

Supplementary materials are available at *Open Forum Infectious Diseases* online. Consisting of data provided by the authors to benefit the reader, the posted materials are not copyedited and are the sole responsibility of the authors, so questions or comments should be addressed to the corresponding author.
