# Supplementary material for: Impact of Rapid Molecular Diagnostic Testing on Outcomes of Patients With Vancomycin-Resistant Enterococcal Bacteremia
Source: Open Forum Infect Dis. 2025 Dec 12;13(1):ofaf757. doi: 10.1093/ofid/ofaf757 (PMC12771509; doi:10.1093/ofid/ofaf757)
Supplement: ofaf757_Supplementary_Data [file ofaf757_supplementary_data.docx]

**Supplementary Table 1. Antimicrobial therapy administered to patients with VRE bacteremia**

|  | **RMDT**  **(n = 237)** | **No RMDT**  **(n = 340)** | ***P*-value** |
| --- | --- | --- | --- |
| First effective antibiotic administered |  |  | 0.276 |
| Daptomycin | 64 (27.0) | 74 (21.8) |  |
| Linezolid | 162 (68.4) | 242 (71.2) |  |
| Other | 11 (4.6) | 22 (6.5) |  |
| None | 0 (0.0) | 2 (0.6) |  |
| Daptomycin dose in mg/kg (median, IQR) | 6.54 (5.10-8.49) | 6.83 (5.49-8.40) | 0.712 |
| Additional anti-VRE antibiotic administered | 78 (32.9) | 69 (20.3) | 0.001 |
| Course of anti-VRE agent completed prior to discharge | 107 (45.1) | 131 (38.5) | 0.112 |
| **All results listed as n (% of total) unless otherwise noted** | | | |

**Supplementary Table 2. Secondary clinical and microbiological outcomes by leukemia and RMDT status**

|  | **Leukemia** | |  | **No Leukemia** | |  |
| --- | --- | --- | --- | --- | --- | --- |
| **Outcome** | **RMDT**  **(n=95)** | **No RMDT (n=156)** | ***P*-value** | **RMDT**  **(n=142)** | **No RMDT**  **(n=184)** | ***P*-value** |
| In-hospital 30-day mortality | 33/95 (34.7) | 49/156 (31.4) | 0.586 | 42/142 (29.6) | 75/184 (40.8) | 0.037 |
| Time from blood culture collection to susceptibilities/detection of vancomycin-resistant enterococci (median hours, IQR) | 20 (17-21) | 61.5 (53-69) | <0.001 | 20 (17-23) | 62 (56-69) | <0.001 |
| Time from blood culture collection to administration of active antimicrobial therapy^+^ (median hours, IQR) | 21 (18-23) | 25 (19-44.5) | <0.001 | 22 (15-26) | 45 (24-64) | <0.001 |
| **Microbiologic Outcomes** |  |  |  |  |  |  |
| Microbiologic Failure | 3 (3.2) | 9 (5.8) | 0.347 | 12 (8.5) | 7 (3.8) | 0.076 |
| Time to first negative blood culture (median hours, IQR) | 53 (30-85) | 59 (32-119) | 0.709 | 40 (25-72) | 51 (29-87) | 0.063 |
| Follow-up blood cultures positive for VRE | 26 (27.4) | 44 (28.2) | 0.886 | 34 (23.9) | 52 (28.3) | 0.380 |
| **Other Clinical Outcomes** |  |  |  |  |  |  |
| Transfer to ICU within 7 days | 10/81 (12.3) | 24/138 (17.4) | 0.320 | 15/101 (14.9) | 10/101 (9.9) | 0.285 |
| ID consult within 7 days of positive culture | 85/95 (89.5) | 141/156 (90.4) | 0.815 | 103/142 (72.6) | 109/184 (59.2) | 0.013 |
| In-hospital mortality | 41 (43.2) | 58 (37.2) | 0.347 | 51 (35.9) | 85 (46.2) | 0.062 |
| Hospital length of stay post-positive culture (median days, IQR) | 15 (6.7-31.5) | 14.4 (8.3-24.4) | 0.858 | 15.5 (8.2-34.1) | 13.5 (5.1-26.6) | 0.074 |
| Hospital readmission within 30 days | 23 (24.2) | 42 (26.9) | 0.634 | 29 (20.4) | 34 (18.5) | 0.659 |
| Bacteremia relapse within 90 days | 12 (12.6) | 17 (10.8) | 0.677 | 12 (8.4) | 17 (9.2) | 0.804 |
| **All results listed as n (% of total) unless otherwise noted** | | | | | | |

**Supplementary Table 3. Multivariate and Univariate Analysis for 30-day mortality in patients with VRE bacteremia**

|  | **Univariate Analysis** | | | **Multivariate Analysis** | | | |
| --- | --- | --- | --- | --- | --- | --- | --- |
| **Variable** | **Odds Ratio** | **95% CI** | ***P*-value** | **Adjusted**  **Odds Ratio** | **95% CI** | ***P*-value** | |
| RMDT use | 0.81 | 0.57 – 1.15 | 0.231 | 0.80 | 0.55 – 1.19 | | 0.272 |
| Age, per year increase | 0.98 | 0.98 – 1.00 | 0.054 | 0.99 | 0.98 - 1.00 | | 0.086 |
| Non-white or unknown race | 1.57 | 1.10 – 2.23 | 0.012 | 0.67 | 0.45 – 1.00 | | 0.064 |
| Charlson Comorbidity Index, per unit increase | 1.04 | 0.96 – 1.13 | 0.321 | 1.08 | 0.99 – 1.19 | | 0.098 |
| Pitt Bacteremia Score, per unit increase | 1.35 | 1.26 – 1.45 | <0.001 | 1.24 | 1.13 – 1.37 | | <0.001 |
| Severe neutropenia (ANC <500) | 0.93 | 0.66 – 1.31 | 0.688 | 1.42 | 0.95 – 2.11 | | 0.088 |

**Supplementary Table 4. Multivariate and Univariate Analysis for 30-day mortality in non-leukemia patients with VRE bacteremia**

|  | **Univariate Analysis** | | | **Multivariate Analysis** | | |
| --- | --- | --- | --- | --- | --- | --- |
| **Variable** | **Odds Ratio** | **95% CI** | ***P*-value** | **Adjusted**  **Odds Ratio** | **95% CI** | ***P*-value** |
| RMDT use | 0.61 | 0.38 – 0.97 | 0.038 | 0.71 | 0.42 – 1.18 | 0.187 |
| Age, per year increase | 0.99 | 0.98 – 1.00 | 0.199 | 1.00 | 0.98 – 1.01 | 0.604 |
| Non-white or unknown race | 1.54 | 0.97 – 2.46 | 0.066 | 1.39 | 0.81 – 2.36 | 0.23 |
| Charlson Comorbidity Index, per unit increase | 1.06 | 0.96 – 1.16 | 0.254 | 1.10 | 0.98 – 1.23 | 0.100 |
| Pitt Bacteremia Score, per unit increase | 1.36 | 1.24 – 1.48 | <0.001 | 1.40 | 1.27 – 1.54 | <0.001 |
| Severe neutropenia (ANC <500 cells/µL) | 1.86 | 1.07 – 3.22 | 0.028 | 2.79 | 1.48 – 5.26 | 0.001 |
